# Mycorrhizal specificity of fully mycoheterotrophic *Yoania* in Taiwan and China and novel natural abundance stable isotope patterns

**DOI:** 10.1111/plb.70195

**Published:** 2026-03-11

**Authors:** Y.‐I. Lee, F. E. Zahn, Y.‐A. Chiang, C.‐K. Yang, H. Jiang, G. Gebauer

**Affiliations:** ^1^ Department of Life Science National Taiwan University Taipei Taiwan; ^2^ Institute of Ecology and Evolutionary Biology National Taiwan University Taipei Taiwan; ^3^ Laboratory of Isotope Biogeochemistry, Bayreuth Center of Ecology and Environmental Research (BayCEER) University of Bayreuth Bayreuth Germany; ^4^ Department of Forestry National Pingtung University of Science and Technology Neipu Pingtung Taiwan; ^5^ Yunnan Laboratory for Conservation of Rare, Endangered and Endemic Forest Plants, National Forestry and Grassland Administration, Yunnan Academy of Forestry and Grassland Kunming Yunnan People's Republic of China; ^6^ Present address: Department of Ecology of Fungi, Bayreuth Center of Ecology and Environmental Research (BayCEER) University of Bayreuth Bayreuth Germany

**Keywords:** Carbon, hydrogen, mycoheterotrophy, mycorrhiza, Orchidaceae, stable isotopes, *Yoania*

## Abstract

- *Yoania* is a rare achlorophyllous mycoheterotrophic orchid genus distributed across Japan, Taiwan, China, India and Vietnam, associating with wood‐decomposing fungi. Studying mycoheterotrophic plants' mycorrhizal diversity is essential, as they depend entirely on fungi for carbon and nutrients. Here, we studied mycorrhizal interactions and nutrient strategies in three *Yoania* species from Taiwan and China. We hypothesize (H1) that *Physisporinus* associates with the *Yoania* species studied, and (H2) that when this symbiotic relationship alters nutritional patterns, this partnership will result in lower δ^13^C enrichment than in other fully mycoheterotrophic, wood‐decaying‐fungus‐associated orchids.
- High‐throughput DNA sequencing was used to investigate the mycorrhizal fungal communities of three *Yoania* species. In addition, natural stable isotopes (δ^13^C and δ^15^N) were measured in two species, while δ^2^H and δ^18^O were measured in one of them to further evaluate nutrient acquisition strategies.
- In Taiwan, *Yoania japonica* and *Yoania amagiensis* var. *squamipes*, and in China, *Yoania prainii*, all associate with a single *Physisporinus* taxonomic unit, distinct from the *Physisporinus* taxonomic units associated with *Yoania* species in Japan. As a white‐rot fungus, *Physisporinus* may preferentially decompose relatively ^13^C depleted lignin likely explaining the lower ^13^C enrichment of *Yoania* in comparison with other fully mycoheterotrophic orchids associated with wood‐decomposing fungi.
- Our combined molecular and isotopic evidence suggests that the mycoheterotrophic orchid genus *Yoania* employs a nutritional strategy that is most likely linked to the use of lignin by its white‐root fungal partner in forest ecosystems.

## Graphical abstract

Three species of the mycoheterotrophic orchid genus *Yoania* found in Taiwan and China are associated with a single taxonomic unit of white‐rot fungus from the genus *Physisporinus* and exhibit a stable isotope pattern distinct from other fully mycoheterotrophic (FMH) orchids associated with wood‐decaying fungi.

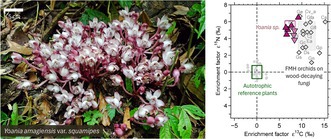

## BACKGROUND

Nearly 90% of land plants form mycorrhizal associations, which play a crucial role in plant nutrient acquisition and ecosystem functioning (Trappe 1987; Wang & Qiu 2006). Mycorrhizal symbiosis is typically mutualistic, where plants supply carbon to fungi in return for essential nutrients, such as phosphorus and nitrogen. A distinctive group of plants, particularly non‐photosynthetic species, have evolved to rely exclusively on mycorrhizal fungi for their nutritional requirements, a strategy referred to as full mycoheterotrophy. These plants depend entirely on their fungal partners for carbon and minerals, representing a unique evolutionary adaptation observed in plant families such as Orchidaceae and Ericaceae (Smith & Read 2008). The orchid family accounts for nearly half of all fully mycoheterotrophic plant species documented to date (Merckx 2013).

In temperate forests, mycoheterotrophic orchids typically associate with specific clades of ectomycorrhizal fungi, enabling them to obtain photosynthates indirectly from neighbouring trees and shrubs through shared underground mycorrhizal networks (Taylor & Bruns 1997; Hynson *et al*. 2013; Merckx *et al*. 2024). In contrast, mycoheterotrophic orchids in tropical, subtropical and warm‐temperate forests in Asia often associate with saprotrophic fungi, including litter‐ and wood‐decomposing fungi, relying on these fungi for carbon and nutrients (Ogura‐Tsujita *et al*. 2009; Lee *et al*. 2015). Studies on these Asian mycoheterotrophic orchids have identified various fungal associations. For instance, *Epipogium roseum* and *Eulophia zollingeri* associate with fungi in the Psathyrellaceae family (Yamato *et al*. 2005; Yagame *et al*. 2007; Ogura‐Tsujita & Yukawa 2008; Suetsugu *et al*. 2024). *Erythrorchis* species form associations with a diverse range of wood‐decomposing fungi from the Hymenochaetaceae and Polyporaceae families (Umata 1995; Dearnaley 2007; Ogura‐Tsujita *et al*. 2018), while *Cyrtosia* and *Galeola* species partner with wood‐decomposing fungi from the Meripilaceae family (Umata *et al*. 2013; Lee *et al*. 2015). Additionally, *Wullschlaegelia aphylla* and several *Gastrodia* species associate with litter‐decomposing fungi from the Mycenaceae and Marasmiaceae families (Martos *et al*. 2009; Ogura‐Tsujita *et al*. 2009; Lee *et al*. 2015; Kinoshita *et al*. 2016; Li *et al*. 2022). Despite their ecological importance for carbon and nutrient cycling in ecosystems, interactions involving mycorrhizal fungi that decay litter and wood remain insufficiently explored.

Analysis of stable isotope natural abundances has been used to study the trophic modes of orchids (Gebauer & Meyer 2003; Gebauer *et al*. 2016; Hynson *et al*. 2016). Tissues of fully mycoheterotrophic orchids are usually enriched in the heavy isotopes ^13^C, ^2^H and ^15^N relative to tissues of autotrophic plants co‐occurring under the same environmental conditions, while plant tissue ^18^O abundance remains unaffected by mycoheterotrophic carbon exchange and rather relates to differences in transpiration (Gebauer *et al*. 2016). The ^15^N signature of mycoheterotrophic orchids differs depending on the source from which mycoheterotrophic orchids gain carbon and thereby enables differentiation of these sources. Orchids that gain carbon from fungi that also form ectomycorrhizas with woody plants are relatively more enriched in ^15^N than orchids that acquire carbon from wood‐ or litter‐decomposing fungi (Martos *et al*. 2009; Ogura‐Tsujita *et al*. 2009, 2018; Motomura *et al*. 2010; Lee *et al*. 2015, 2025; Suetsugu *et al*. 2020, 2022, 2024; Yagi *et al*. 2024; Suetsugu *et al*. 2025; Suetsugu & Okada 2025a, 2025b). Yet, for orchids associated with wood‐ or litter‐decomposing fungi, data on stable isotope natural abundances are scarce.

*Yoania* is a genus of mycoheterotrophic orchids native to India, southern China, Japan, Taiwan and Vietnam (POWO 2024). In Taiwan, two *Yoania* species, *Y. japonica* and *Y. amagiensis* var. *squamipes*, are found in forests with decomposing logs and typically bloom in summer (Chang & Lin 1990). These species are also found in Japan, indicating a shared regional distribution. During a field trip to southeastern Yunnan, China, *Y. prainii* individuals were observed growing near a decaying broad‐leaved tree. These observations suggest that *Yoania* species consistently prefer habitats with abundant decomposing wood. In temperate deciduous forests of Japan, *Yoania* species predominantly associate with *Physisporinus*, a wood‐decomposing white‐rot fungus (Yamashita *et al*. 2020), suggesting that it serves as the primary fungal partner for these orchids. This raises the question of whether *Physisporinus* also dominates the mycorrhizal fungal communities of *Yoania* species in Taiwan and China, or if different fungal partners are involved in these regions. In this study, we examined the mycorrhizal fungal communities using high‐throughput DNA sequencing. To confirm the mycoheterotrophic nature of *Yoania* species and elucidate their pathways for nutrient acquisition, we analysed the natural abundances of ^13^C, ^15^N, ^2^H and ^18^O stable isotopes whenever sufficient sample material was available.

We expect (H1) *Physisporinus* to be the fungal partner of the *Yoania* species investigated from Taiwan and China, and when this symbiotic relationship alters nutritional patterns, (H2) a lower ^13^C enrichment of *Yoania* relative to the other so far investigated fully mycoheterotrophic orchids associated with wood‐decomposing fungi caused by its carbon acquisition from a white‐rot fungus.

## METHODS

### Study sites and mycorrhiza sampling

**Fig. 1:**
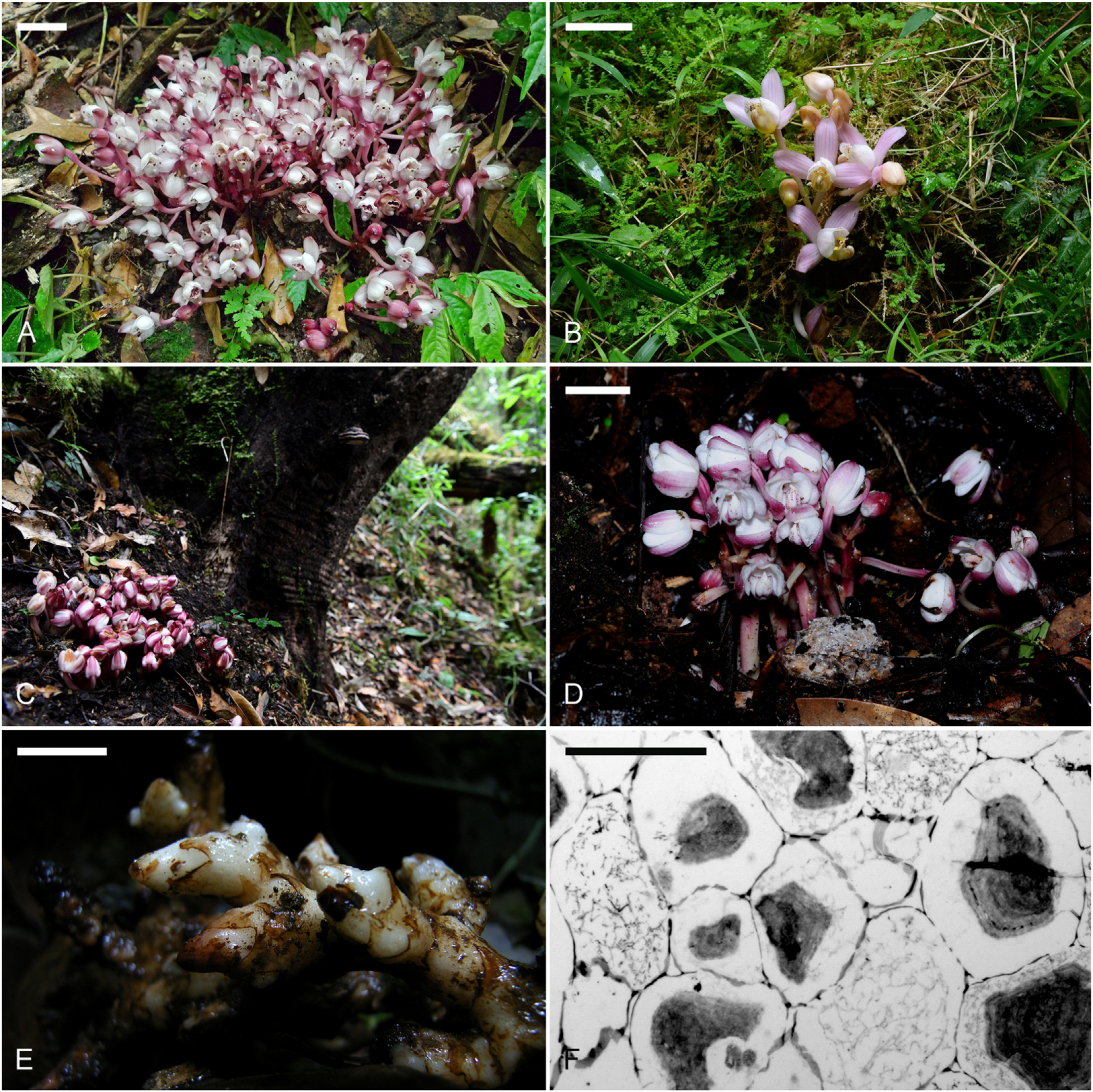
The morphology and rhizome section of *Yoania* species. (A) *Y. amagiensis* var. *squamipes*, bar = 5 cm; (B) *Y. japonica*, bar = 5 cm; (C) Habitat of *Y. prainii*. (D) *Y. prainii*, bar = 5 cm; (E) The rhizome of *Y. amagiensis* var. *squamipes*, bar = 1 cm; (F) Cross section of a mycorrhizal rhizome of *Y. amagiensis* var. *squamipes*, showing fungal colonization in the inner cortical parenchyma cells, bar = 50 μm.

The study site of *Yoania amagiensis* var. *squamipes* (Fig. 1A) was located in a forest primarily composed of *Cryptomeria japonica* (a species introduced from Japan during the early 20th century) and *Phyllostachys edulis* (an introduced bamboo species commonly managed in plantations) along the Rueytei Ancient Path in Chiayi County, southern Taiwan (23°32′7.3″N, 120°41′29″E), at an elevation of 1200 m a.s.l. The study site of *Y. japonica* (Fig. 1B) was located in a mixed forest consisting of native Fagaceae interspersed with *Cryptomeria japonica* and *Chamaecyparis formosensis* at an elevation of 1600 m a.s.l. in Taipingshan, Ilan County, northeastern Taiwan (24°30′28″N, 121°36′43″E). The study site of *Y. prainii* (Fig. 1C and D) was located in a broadleaf forest in Daweishan, Honghe County, Yunnan Province, China at 1100 m a.s.l. (23°0′43″N, 104°49′45″E). Voucher specimens of *Y*. *japonica* (YIL201506) and *Y. amagiensis* var. *squamipes* (YIL201507) were deposited in the herbarium of National Museum of Natural Science, Taichung, Taiwan. Voucher specimens of *Y. prainii* (YAF16321) were deposited in the herbarium of Yunnan Academy of Forestry and Grassland, Kunming.

The sample size of *Yoania* species was limited due to their rarity and small population sizes. Initially, five individuals of *Y. japonica*, three individuals of *Y*. *amagiensis* var. *squamipes* and three individuals of *Y*. *prainii* were collected from 1 m^2^ plots, each containing a single adult orchid. Fungal colonization was assessed in rhizomes using free‐hand sections observed under a light microscope. Due to the limited number of rhizomes showing colonization, the final samples examined included *Y. japonica* (n = 3), *Y. amagiensis* var. *squamipes* (n = 2) and *Y. prainii* (n = 3), yielding a total of eight study samples.

### Microscopy

Mycorrhizal rhizomes were fixed in 2.5% glutaraldehyde buffered with 0.1 M phosphate buffer at 4 °C overnight. Following fixation, the samples were washed three times with 0.1 M phosphate buffer, dehydrated through a graded ethanol series and embedded in Technovit 7100 (Kulzer & Co., Wehrheim, Germany). Sections, 3 μm thick, were prepared using glass knives on a Leica RM2125 rotary microtome. These sections were stained with the Periodic acid – Schiff (PAS) reaction to detect total insoluble carbohydrates and counterstained with 0.05% (w/v) toluidine blue O (TBO), following the protocol of Lee *et al*. (2015). The stained sections were examined, and images were captured using a digital camera mounted on an Axioskop 2 microscope (Carl Zeiss AG, Jena, Germany).

### Molecular identification of mycorrhizal fungi using high‐throughput sequencing

Mycorrhizal rhizomes (Fig. 1E and F) were surface sterilized with 1% sodium hypochlorite for 2 min, rinsed three times for 3 min each with sterile distilled water and stored at −80 °C until further use. DNA was extracted from each sample using the DNeasy Plant Mini Kit (Qiagen, Hilden, Germany). The ITS1 region of the nuclear ribosomal RNA genes was amplified using the primer pair ITS1F and ITS2R (Adams *et al*. 2013). PCR reactions contained 20 μl in total, including 10 ng of genomic DNA template, 0.8 μl of each primer (5 μM), 2 μl of 2.5 mM dNTPs, 4 μl of 5× TransStart^®^FastPfu Buffer (TransGen Biotech Co., Ltd., Beijing, China) and 0.4 μl of TransStart^®^FastPfu Polymerase. The parameters of reactions consisted of an initial denaturation at 95 °C for 3 min, then 35 cycles of 95 °C for 30 s and 55 °C for 30 s, and a final extension of 72 °C for 45 s. PCR products were separated by gel electrophoresis and amplicons within the appropriate size range were cut and purified using the AxyPrep DNA Gel Extraction Kit (Axygen Biosciences, Union City, CA) and quantified using QuantiFluor™ Fluorometer (Promega Corporation, Madison, WI). After library construction, the samples were pooled at equimolar concentrations and sequenced using the Illumina MiSeq PE300 system at Genomics BioSci & Tech Co., Ltd. (Taipei, Taiwan).

Raw data were demultiplexed and quality‐filtered using the QIIME pipeline (version 1.17). We applied a quality filtering protocol where 300 bp reads were pruned if the average quality score dropped below 20. Additionally, sequences containing ambiguous ‘N’ bases or those shorter than 50 bp were discarded. Only sequences with an overlap longer than 10 bp were merged based on their overlapping regions, while reads that could not be assembled were excluded. For operational taxonomic units (OTUs) construction, we employed the UPARSE algorithm (USEARCH v7; Edgar 2013) at a 97% identity threshold. Chimeric sequences were filtered out utilizing the UCHIME reference dataset from UNITE. Taxonomic assignment for each ITS1 region was conducted via the RDP Classifier (http://rdp.cme.msu.edu/), referencing the UNITE fungal database with a 70% confidence cut‐off. Taxonomic identities of the remaining OTUs were determined using the BLAST analysis (Altschul *et al*. 1990) of OTU representative sequences against the GenBank database (Benson *et al*. 2009). The identified OTUs were then manually screened to identify potential orchid‐associated mycorrhizal families following a stepwise approach, discarding those with short sequences (<150 bp) or low sequence similarity (<90%) to fungal species across their entire sequence length, as well as those represented by a single sequence in each orchid root sample. The results were compiled into an OTU table, with each cell indicating read numbers (Table S1).

### Phylogenetic analyses

The nuclear internal transcribed spacer (ITS) region of the mycorrhizal fungi was amplified with the fungal specific primer sets ITS1F/ITS4 (White *et al*. 1990; Gardes & Bruns 1993). PCR amplification and sequencing were carried out as described by Lee *et al*. (2015). GenBank accession number of ITS sequence was PX106494. Sequences of *Physisporinus* species (Meripilaceae) and *Phlebia* species (Meruliaceae) were downloaded from the NCBI database (National Center for Biotechnology Information, GenBank) by referring to Yamashita *et al*. (2020) and Zhao *et al*. (2023). These sequences were aligned using MAFFT v7.526 with the L‐INS‐i algorithm (Katoh & Standley 2013). *Trametes versicolor* was chosen as outgroup taxon to root the tree. Poorly aligned regions were removed using trimAl v1.4.15 with the default ‘automated1’ setting (Capella‐Gutiérrez *et al*. 2009). The substitution model GTR + F + I + G4 was identified as the best‐fit model based on the Bayesian Information Criterion (BIC) using ModelFinder, implemented in IQ‐TREE v2.2.2.6 (Minh *et al*. 2020). Bayesian phylogenetic inference was conducted in MrBayes v3.2.7 (Ronquist *et al*. 2012), with a Markov Chain Monte Carlo (MCMC) analysis run for 1,500,000 generations, sampling every 1,000 generations. Convergence was confirmed by ensuring the final average standard deviation of split frequencies was below 0.01. The first 25% of sampled trees were discarded as burn‐in. The resulting phylogenetic tree was visualized and annotated in FigTree v1.4.4 (Rambaut 2018), with posterior probabilities used to assess branch support.

### Stable isotope natural abundance and nitrogen concentration analyses

For natural abundance stable isotope (δ^13^C, δ^15^N, δ^2^H, δ^18^O) and nitrogen concentration analyses, we used a reference system sampling approach as described by Gebauer & Meyer (2003). In short, we selected 1 m^2^ plots, each containing one adult orchid species individual and three autotrophic reference plant species. We sampled flower stalks of *Y. japonica* and *Y. amagiensis* var. *squamipes* individuals at 5 and 3 plots, respectively, together with three autotrophic reference plants per plot (Table S2). *Y. prainii* samples were unavailable for isotope analysis. Samples were oven‐dried to constant weight at 105°C, ground to fine powder in a ball mill (Retsch Schwingmühle MM2, Haan, Germany) and stored in a desiccator until analyses.

For relative natural abundance analysis of carbon (^13^C/^12^C) and nitrogen (^15^N/^14^N) isotopes as well as nitrogen concentrations, samples of *Y. japonica* and *Y. amagiensis* var. *squamipes* and reference plant material were weighed into tin capsules (plant sample weight: 1–2 mg) using micro balances (Sartorius CPA2P & MSE3.6P‐000‐DM, Göttingen, Germany & Mettler AT21, Gießen, Germany). Analyses were carried out simultaneously using an EA‐IRMS coupling combining an elemental analyser (CE Instruments 1108, Milano, Italy) with a continuous flow isotope ratio mass spectrometer (delta S, Finnigan MAT, Bremen, Germany) via a ConFlo III open‐split interface (Finnigan MAT, Bremen, Germany).

For *Y. amagiensis* var. *squamipes*, sample material was sufficient to additionally determine relative natural abundances of hydrogen (^2^H/^1^H) and oxygen isotopes (^18^O/^16^O) separately using a TC‐IRMS coupling, which links a thermal conversion through a pyrolysis unit (HTO, HEKAtech, Wegberg, Germany) to a continuous flow isotope ratio mass spectrometer (delta V advantage, Thermo Fisher Scientific, Bremen, Germany) via a ConFlo IV open‐split interface (Thermo Fisher Scientific, Bremen, Germany). In this case, samples were weighed into annealed silver capsules (plant sample weight: 0.5–1 mg) and analysed as described by Gebauer *et al*. (2016).

Measured relative isotope abundances were calculated according to the following equation: δ^13^C, δ^15^N, δ^2^H or δ^18^O = (R_sample_/R_standard_ – 1) × 1,000 [‰], where R_sample_ and R_standard_ are the ratios of heavy to light isotope of the samples and the respective standard. Standard gases were calibrated in relation to international standards [CO_2_ *versus* V‐PDB (R = 0.0111802), N_2_ *versus* N_2_ in air (R = 0.0036765), H_2_ *versus* V‐SMOW (R = 0.00015575) and CO *versus* V‐SMOW (R = 0.0020052)]. Reference substances were ANU sucrose and NBS19 for C isotopes, N1 and N2 for the N isotopes, IAEA‐CH7, V‐SMOW and SLAP for H isotopes, and IAEA601 and IAEA602 for O isotopes, all provided by the International Atomic Energy Agency, Vienna, Austria. Acetanilide and Benzoic acid served as quality control for C and N isotope and H and O isotope abundance measurements, respectively, and for element concentration calculations. We then normalized the measured δ values for time‐ and site‐differences according to Preiss & Gebauer (2008), calculating enrichment factors (ε): ε = δ_S_ − δ_REF_, where δ_S_ is a single δ^13^C, δ^15^N, δ^18^O or δ^2^H value of an orchid individual and δ_REF_ is the mean value of all autotrophic reference plants within the same plot.

We tested for pairwise differences in the isotopic enrichment factors ε^13^C, ε^15^N, ε^2^H and ε^18^O and nitrogen concentration between the sampled *Yoania* species and autotrophic reference plants using non‐parametric Mann–Whitney *U*‐tests with Bonferroni–Holm correction. In addition, we compared the ε^13^C and the ε^15^N of our *Yoania* samples with the ε^13^C and the ε^15^N of fully mycoheterotrophic orchid species that are known to be mycorrhizal with wood‐decaying fungi extracted from the literature using a non‐parametric Mann–Whitney *U*‐test. For statistical analyses, we used *R* version 4.4.0 (R Development Core Team 2024) with a significance level of α = 0.05.

## RESULTS

### Fungal identification

**Fig. 2:**
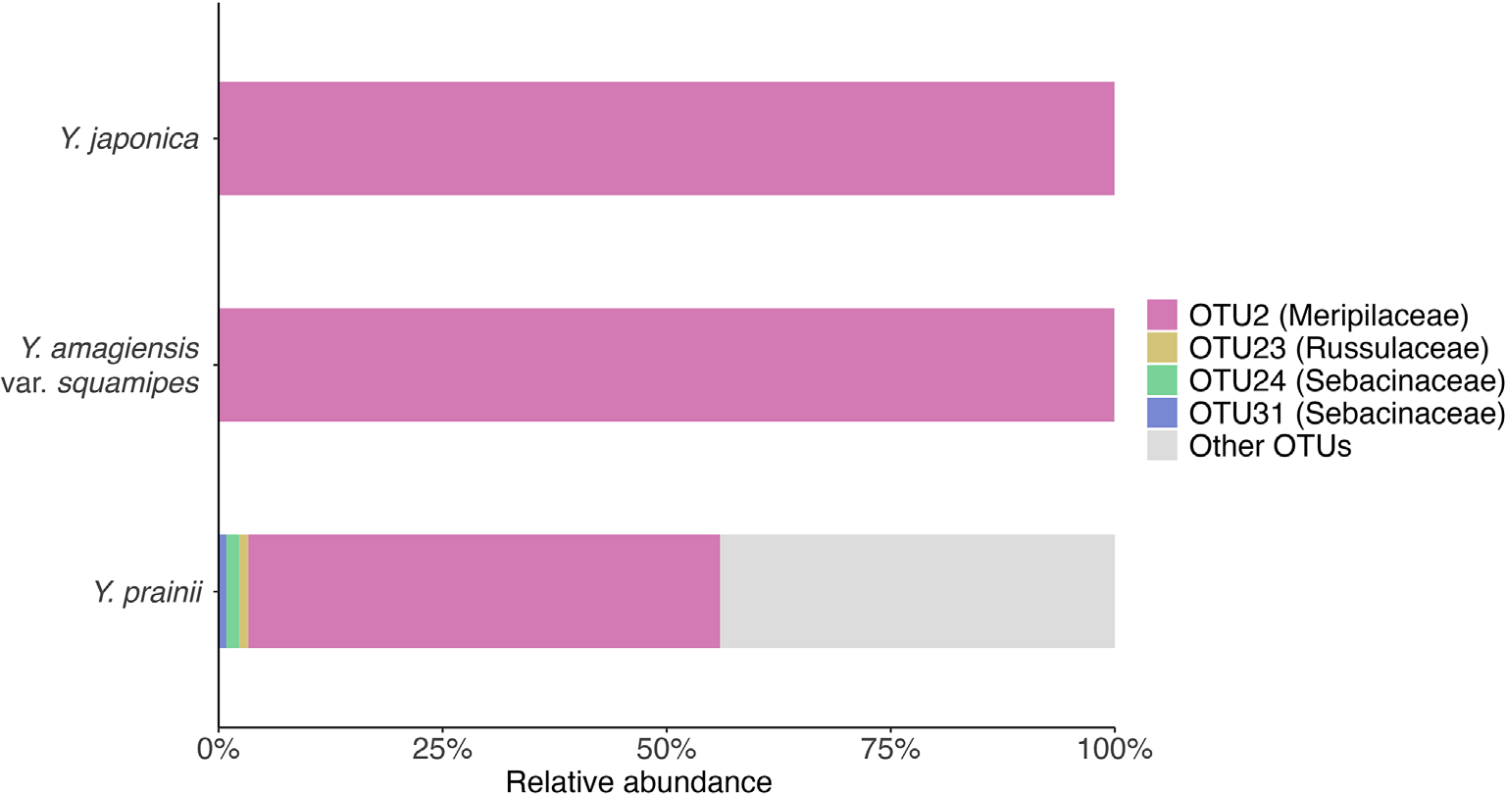
Relative abundance of orchid mycorrhizal fungi amplified from *Yoania prainii*, *Y. japonica* and *Y. amagiensis* var. *squamipes* at the family level.

From eight rhizomes samples of three *Yoania* species, fungal ITS sequences were acquired. After quality filtering, we obtained 290,505 reads. BLAST search revealed that the three *Yoania* species studied were predominantly associated with the genus *Physisporinus* of Meripilaceae (OTU2 – 254,866 reads, 87.73%) (Fig. 2 and Table S1). Using BLAST analysis, the ITS sequence of this OTU showed similarity to several *Physisporinus* species and a *Phlebia* strain (KJ140617) collected from maple‐dominated forests in Wisconsin (Brazee *et al*. 2014). In *Y. japonica* and *Y. amagiensis* var. *squamipes*, more than 99.9% reads were assigned to this OTU. However, in *Y. prainii*, a large proportion of reads were assigned to OTUs that are not putative mycorrhizal fungi (32,815 reads; 44.0%). Among these, a substantial number of reads belonged to *Neonectria* (OTU3 – 27,551 reads; 29.9%). Although three OTUs belonging to putative orchid mycorrhizal fungi, specifically Russulaceae and Sebacinaceae, were identified, only a few reads (2690 reads; 0.93%) were recorded for these fungi.

**Fig. 3:**
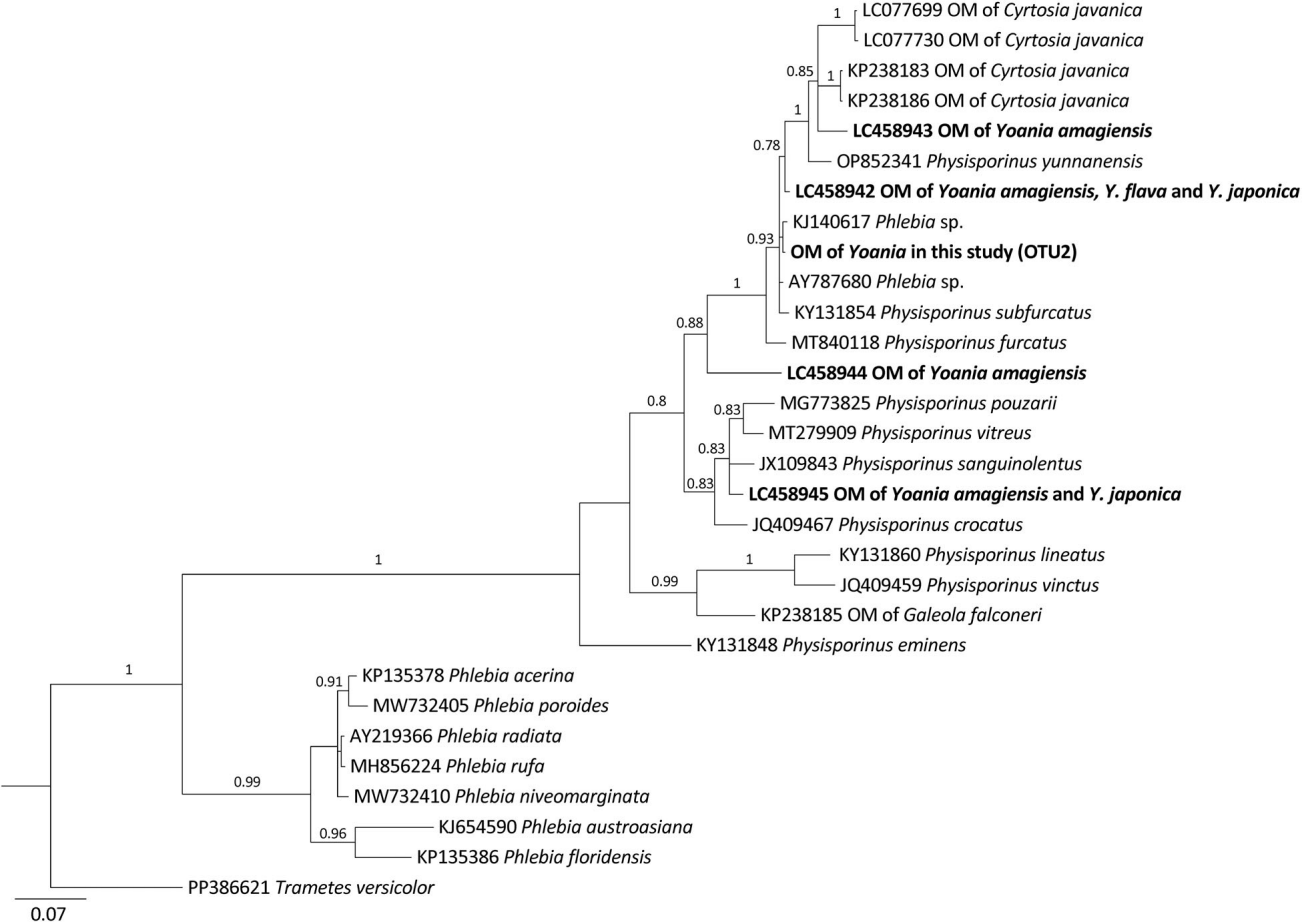
Phylogenetic relationship of mycorrhizal fungi associated with *Yoania amagiensis* var. *squamipes, Y. japonica* and *Y. prainii* based on the rDNA ITS sequences of *Phlebia* and *Physisporinus* available in GenBank. The analysis was conducted using the Maximum Likelihood method with 1,000 bootstrap replicates.

To further characterize the mycorrhizal fungi associated with these *Yoania* species, a Bayesian phylogenetic tree was constructed based on the predominant mycorrhizal fungi in three *Yoania* species (OTU2), sequences from other orchid mycorrhizal (OM) fungi previously identified from *Yoania* species from Japan, and closely related *Physisporinus* and *Phlebia* sequences obtained from GenBank (Fig. 3). The resulting tree revealed that the predominant mycorrhizal fungi of three *Yoania* species in Taiwan and China formed a well‐supported monophyletic clade with *Physisporinus* species.

### Stable isotope patterns

**Fig. 4:**
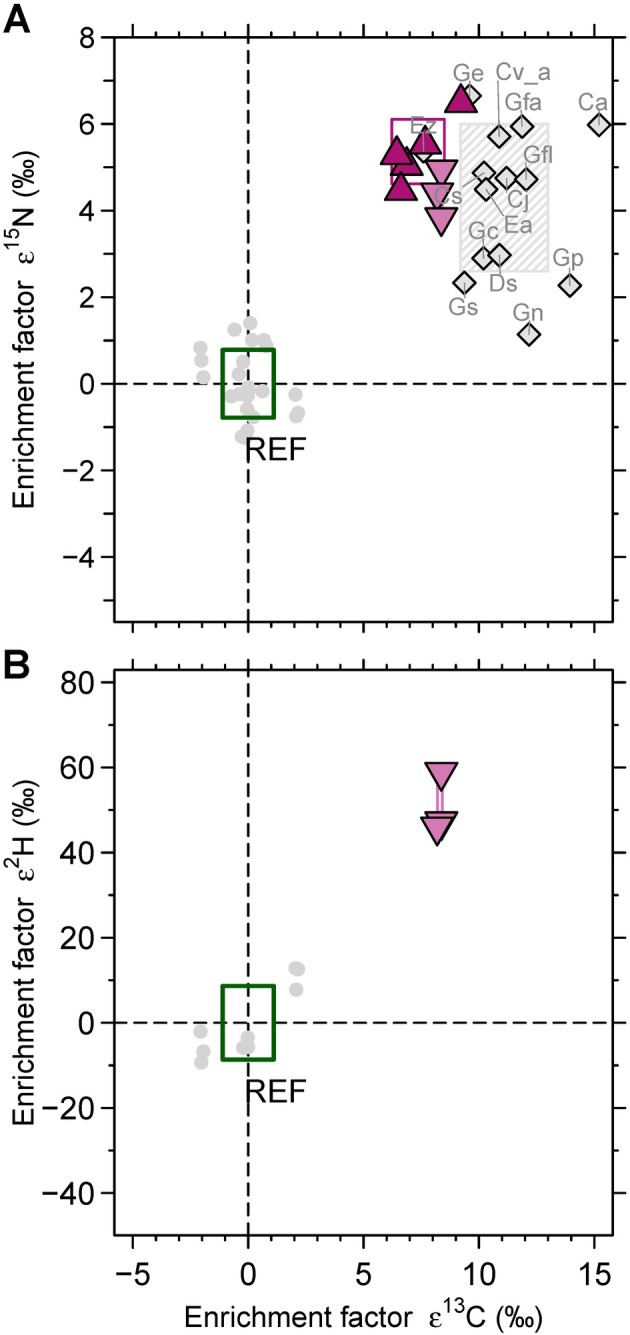
Enrichment factors (A) ε^15^N, ε^13^C and (B) ε^2^H, ε^13^C (symbols) ± 1 SD (frames) as calculated for *Yoania amagiensis* var. *squamipes* (n = 3, downwards triangles), *Y. japonica* (n = 5, upwards triangles) and co‐occurring photosynthetic reference plants (grey dots, green frame, n = 24) from Taiwan, as well as for fully mycoheterotrophic orchids (n_samples_ = 58, n_species_ = 13) known to be mycorrhizal with wood‐decomposing fungi based on literature data. Literature records include *Gastrodia similis* (Gs), *Gastrodia confusa* (Gc), *Gastrodia flabilabella* (Gfl), *Gastrodia elata (*Gl), *Gastrodia nipponica* (Gn), *Galeola falconeri* (Gfa), *Cyrtosia septentrionalis* (Cs), *Cyrtosia javanica* (Gj), *Erythrorchis altissima* (Ea), *Cremastra aphyla* (Ca), albino *Cremastra variabilis* (Cv_a), *Eulophia zollingeri* (Ez) and *Didymoplexis siamensis* (Ds). Data source: Martos *et al*. (2009); Ogura‐Tsujita *et al*. (2009, 2018); Motomura *et al*. (2010); Lee *et al*. (2015, 2025); Suetsugu *et al*. (2020, 2022, 2024); Suetsugu *et al*. (2025); Suetsugu & Okada (2025a).

Flower stalks of both *Yoania* species sampled in Taiwan were similarly enriched in ^13^C and ^15^N when compared to co‐occurring autotrophic reference plant leaves (Fig. 4 and Table S3): *Y. japonica* – ε^13^C = 7.36 ± 1.14‰ (*P* < 0.001, effect size of 0.643), ε^15^N = 5.36 ± 0.74‰ (*P* = 0.001, effect size of 0.643) and *Y. amagiensis* var. *squamipes* – ε^13^C = 8.31 ± 0.10‰ (*P* = 0.009, effect size of 0.535), ε^15^N = 4.40 ± 0.56‰ (*P* = 0.009, effect size of 0.535). For a small sample size of *Y. amagiensis* var. *squamipes* and reference plants, we additionally obtained ^2^H and ^18^O data (Fig. 4B and Fig. S1B): *Y. amagiensis* var. *squamipes* was by 50.73 ± 7.08‰ significantly enriched in ^2^H relative to surrounding reference plants (*P* = 0.009, effect size of 0.721), while enrichment in ^18^O (1.21 ± 0.22) was not significantly distinct from reference plants (*P* = 0.727, effect size of 0.133) (Table S3). For both *Yoania* species, the nitrogen concentration of the samples was higher than that of the reference plants at the same site (Fig. S1A). However, only for *Y. amagiensis* var. *squamipes*, the higher nitrogen concentration was significant (*P* = 0.018, effect size of 0.667) (Table S3).

Comparing our two *Yoania* species (n = 8) with the group of fully mycoheterotrophic orchids on wood‐decaying fungi from the literature (n = 62), they displayed significantly lower ε^13^C (*P* = 0.001) with a moderate effect size of 0.384, while ε^15^N did not differ (*P* = 0.249, effect size of 0.139) (Fig. 4A and Table S4).

## DISCUSSION

### Physisporinus as the fungal partner of *Yoania* species

Our results revealed that *Yoania* species from Taiwan and China primarily associated with a single *Physisporinus* OTU (Table S1). Although this clade also includes two strains identified as *Phlebia* (KJ140617 and AY787680) from NCBI database, the strain grouped with strong support (Bayesian posterior probability = 0.93) alongside *Physisporinus* (Meripilaceae), rather than with other *Phlebia* species (Meruliaceae), raising questions about the taxonomic placement of these two *Phlebia* strains (Fig. 3). Similarly, Kondratyuk *et al*. (2015) noted that strain KJ140617 is phylogenetically distinct from typical *Phlebia* species and more closely related to *Physisporinus*.

In temperate regions, fungal specificity of mycoheterotrophic plants is reported to be generally higher than that of photosynthetic plants (Leake 1994; Bidartondo & Bruns 2002; Taylor *et al*. 2003). However, mycoheterotrophic orchids in Asia exhibit a spectrum of fungal specificity. Some species display low specificity, such as *Aphyllorchis* species, which associate with multiple ectomycorrhizal families (Roy *et al*. 2009), and *Erythrorchis altissima*, which forms associations with a broad range of wood‐decaying fungi (Ogura‐Tsujita *et al*. 2018). On the other hand, others exhibit high specificity, including *Eulophia zollingeri*, which exclusively associates with the *Psathyrella candolleana* species group (Ogura‐Tsujita & Yukawa 2008).

Compared to the findings in Japan (Yamashita *et al*. 2020), our result also demonstrated high fungal specificity and notable regional variation in the fungal associations of *Yoania* species. In Japan, *Y. flava* and *Y. japonica* each associate with a single *Physisporinus* OTU, while *Y. amagiensis* associates with four distinct OTUs. In Taiwan, *Y. japonica* and *Y. amagiensis*, and in China, *Y. prainii*, all associate with a single *Physisporinus* OTU, which differs from those in Japan. *Y. japonica* and *Y. amagiensis* occur in both Taiwan and Japan, yet their associated OTUs differ between the regions. The regional divergence in fungal associations is likely influenced by variations in vegetation composition between temperate and subtropical forests, which may result in differences in fungal community structure within the substrate. Polyporales, as primary decomposers of freshly fallen logs or standing deadwood, often dominate the fungal community during the early to mid‐stages of wood decay in both temperate and tropical forests (Renvall 1995; Stockland *et al*. 2012; Purahong *et al*. 2024). Among them, *Physisporinus* is a white‐rot fungus, characterized by its ability to decompose lignin efficiently, the most recalcitrant component of the plant cell wall (Dai 2012; Del Cerro *et al*. 2021). Remarkably, certain *Physisporinus* species can colonize rotten wood even in freshwater environments (Shino *et al*. 2022). In addition, some species of this genus form synnema‐like structures, which are thought to enhance their ability to decay wet wood under humid, oxygen‐limited conditions typical of subtropical forest understories (Shino *et al*. 2023), which is particularly relevant in our study sites. The efficient lignin decomposition by *Physisporinus* likely creates a carbon‐rich microhabitat that supports *Yoania* in the nutrient‐poor and shaded environments. *Physisporinus* has also been identified as a mycorrhizal partner for other fully mycoheterotrophic orchids in the genera *Cyrtosia* and *Galeola* (Umata *et al*. 2013; Lee *et al*. 2015). While *Cyrtosia* and *Galeola* belong to the subfamily Vanilloideae, *Yoania* is classified in the phylogenetically distant subfamily Epidendroideae. The repeated association of *Physisporinus* with these distantly related orchid subfamilies suggests convergent evolution in mycorrhizal symbioses, possibly driven by similar ecological pressures or selective forces (Yamashita *et al*. 2020). The ability of *Physisporinus* to colonize and decompose woody substrates may provide a distinct advantage for nutrient acquisition, enabling these mycoheterotrophic orchids to thrive in resource‐limited forest understories.

### Stable isotope signatures of mycoheterotrophic orchids associated with white‐rot mycobiont Physisporinus

Fully mycoheterotrophic *Yoania* species displayed natural abundance carbon and nitrogen stable isotope signatures within the range known for achlorophyllous orchids gaining organic matter from wood‐ or litter‐decaying fungi (Table 1). Due to a considerably lower ^15^N enrichment, fully mycoheterotrophic orchids on litter‐ or wood‐decaying fungi are clearly distinguished from fully mycoheterotrophic orchids associated with ectomycorrhizal fungi (Table 1). Furthermore, the so far investigated fully mycoheterotrophic orchids on wood‐decaying fungi are more enriched in ^13^C than fully mycoheterotrophic orchids associated with litter‐decaying or ectomycorrhizal fungi (Table 1). Notably, the ^13^C signature of the here investigated wood‐decaying *Yoania amagiensis* var. *squamipes* and *Y. japonica* individuals was low in comparison to previously investigated fully mycoheterotrophic orchids on wood‐decaying fungi. The ^13^C signature of both *Yoania* species only overlapped with the ^13^C signature of *Eulophia zollingeri* – an orchid species that may not be fully mycoheterotrophic and thus less enriched in ^13^C compared to fully mycoheterotrophic orchids on wood‐decaying fungi (Suetsugu *et al*. 2024).

**Table 1:** Mean enrichment factors ɛ^15^N, ɛ^13^C and ɛ^2^H *±* SD of adult leaves of *Yoania amagiensis var. squamipes and Yoania japonica* (study species) and Orchidaceae specimens extracted from published literature until December 2025 for comparison. Comparative values of orchid specimens are grouped by their type of fungal partner: Association of fully mycoheterotrophic (FMH) achlorophyllous orchid species with saprotrophic wood‐ or litter‐decomposing fungi (SAP wood/ litter) and with ectomycorrhizal fungi of trees (ECM).

	ɛ^15^N				ɛ^13^C				ɛ^2^H			
	n_samples_	n_species_	mean	SD	n_samples_	n_species_	mean	SD	n_samples_	n_species_	mean	SD
*Yoania amagiensis* var. *squamipes*	3	1	4.40	0.56	3	1	8.31	0.10	3	1	50.73	7.08
*Yoania japonica*	5	1	5.36	0.74	5	1	7.36	1.14	NA	NA	NA	NA
FMH SAP wood	62	14	4.25	2.00	62	14	10.75	2.42	NA	NA	NA	NA
FMH SAP litter	35	5	2.63	2.47	35	5	8.13	1.50	20	2	51.42	8.90
FMH ECM	175	15	11.86	3.22	175	15	7.83	1.57	10	1	57.30	13.83

*Note:* Data as compiled in Zahn *et al*. (2022), updated by data from Yagi *et al*. (2024); Suetsugu *et al*. (2024, 2025); Suetsugu & Okada (2025a, 2025b); Lee *et al*. (2025).

Many wood‐saprotrophic fungi have been classified into either brown‐rot and soft‐rot fungi preferentially decomposing cellulose and hemicellulose, or white‐rot fungi gradually decomposing lignin (unspecific) and hemicellulose (specific) while crystalline cellulose remains (Floudas 2021). To the best of our knowledge, the here presented ^13^C, ^15^N, ^2^H and ^18^O natural abundance data of *Yoania* is the first comparative dataset obtained from orchids exclusively associated with white‐rot fungal partners and co‐occurring reference plants. In comparison to available natural abundance stable isotope data on mycoheterotrophic orchids with a range of unspecified wood‐decaying fungal partners (Fig. 4A, Martos *et al*. 2009; Ogura‐Tsujita *et al*. 2009, 2018; Motomura *et al*. 2010; Lee *et al*. 2015, 2025; Suetsugu *et al*. 2020, 2022, 2024; Suetsugu *et al*. 2025; Suetsugu & Okada 2025a, 2025b), *Yoania* was significantly less enriched in ^13^C while the ^15^N signature was not distinguished (Table S4). We argue that this pattern likely emerged due to the fact that white‐rot fungi selectively decompose lignin from dead wood. Challenging the traditional view that white‐rot fungi only degrade lignin outside their cells and do not use carbon derived from lignin for their own cellular processes, recent evidence suggests that white‐rot fungi can indeed break down and use lignin carbon internally (Del Cerro *et al*. 2021). Lignin is by about 2.6‰ depleted in ^13^C compared to bulk tree wood and by about 3.4‰ depleted in ^13^C compared to wood cellulose and hemicellulose (Benner *et al*. 1987). The ^13^C depletion of lignin and the ^13^C enrichment of cellulose and hemicellulose in comparison to plant bulk tissue can be traced back to isotope discrimination between primary and secondary plant products (Benner *et al*. 1987; Gleixner *et al*. 1993). Thus, the ^13^C depletion found for *Yoania* in comparison to other so far investigated fully mycoheterotrophic orchids on wood‐decaying fungi can most likely be traced back to the ^13^C‐depleted substrate lignin selectively utilized by its white‐rot fungal host. The substrate of fungal hosts has also been previously reported as a driver for the isotope signature of mycoheterotrophic orchids for the carbon isotope signature of rhizoctonia‐mycorrhizal species (Gebauer *et al*. 2016) and for the nitrogen isotope signature of ectomycorrhizal‐associated species (Schiebold *et al*. 2017). The isotopic values may also reflect the characteristics of locally available woody substrates. Relatively low ^13^C enrichments similar to those reported here have also been observed in the protocorms of *Cremastra* and *Oreorchis patens* (Zahn *et al*. 2022; Suetsugu & Okada 2025c). By contrast, in *Cremastra aphylla*, which associates with a similar fungal group, very high ^13^C enrichment has been recorded (Suetsugu *et al*. 2022). This suggests that the degree of ^13^C enrichment may be shaped not solely by fungal lineage and its decomposition ability but also by the condition of the substrates being utilized.

In contrast to the mostly host–substrate‐driven carbon and nitrogen stable isotope signatures of fully mycoheterotrophic orchids, their hydrogen and oxygen isotope signatures are likely process‐driven. All so far investigated fully mycoheterotrophic orchids, irrespective of whether associated with ectomycorrhizal fungi or with litter‐decaying fungi or in the case of *Yoania amagiensis* var. *squamipes* with white‐rot wood‐decaying fungi (Table 1), are fairly similar in their hydrogen isotope enrichment in comparison to autotrophic plants. The longer the metabolic chain from primarily ^2^H depleted carbohydrates of photosynthetic plants *via* either mycorrhizal or saprotrophic, but in any case, heterotrophic fungi towards fully mycoheterotrophic plants is, the more organic compounds become enriched in the heavy isotope ^2^H (Gebauer *et al*. 2016). The database of hydrogen isotope enrichment factors among mycoheterotrophic plants is still rather limited. Further investigations are urgently required to test whether our current knowledge can be confirmed. In contrast to the significant enrichment in the heavy hydrogen isotope ^2^H found for the various groups of mycoheterotrophic orchids, neither *Yoania amagiensis* var. *squamipes* nor fully mycoheterotrophic orchids associated with ectomycorrhizal fungi or litter‐decaying fungi were markedly distinguished in their oxygen isotope signature from accompanying autotrophic plants. Thus, differences in transpiration can be ruled out as drivers for the significant ^2^H enrichment found for the fully mycoheterotrophic orchids. Differences in transpiration would cause a simultaneous shift of ^18^O and ^2^H in the same direction (Sternberg 1988).

In agreement with many other previously investigated fully and partially mycoheterotrophic orchids (*e.g*. Gebauer & Meyer 2003; Hynson *et al*. 2013, 2016) both here investigated *Yoania* species turned out to have higher total nitrogen concentrations than neighbouring autotrophic plants. This pattern distinguishes mycoheterotrophic orchids from mycoheterotrophic Ericaceae (Hynson *et al*. 2016). The high nitrogen concentrations observed in mycoheterotrophic orchids likely reflect their distinct nitrogen‐transfer mechanism, where mass flow of nitrogen derived from digested fungal tissues (pelotons) provides greater nitrogen input than the physiologically different exchange occurring in Ericaceae, which rely on specialized penetrated epidermal cells as their mycorrhizal interface (Hynson *et al*. 2016).

## CONCLUSIONS

*Yoania* species primarily occur in broad‐leaved forests with abundant decaying deadwood in subtropical East Asia. This study provides the first report of *Yoania* mycorrhizal association combining molecular identification and stable isotope analyses. We identified that *Y. japonica* and *Y. amagiensis* var. *squamipes* from Taiwan and *Y. prainii* from China are predominantly associated with a single OTU belonging to the white‐rot fungus *Physisporinus*, Meripilaceae, exhibiting high fungal specificity and showing no significant variation among sites and species. The unique stable isotope pattern found for two of the *Yoania* species from Taiwan most likely reflects a nutrient acquisition strategy driven by lignin utilization of their white‐rot fungal partner.

## AUTHOR CONTRIBUTIONS

The study idea was conceived by Y‐IL and GG Sampling was done by C‐KY, Y‐IL and HJ. Y‐IL and Y‐AC carried out the molecular analyses. GG and FEZ analysed the isotope abundance data. Y‐IL, GG, Y‐AC and FEZ wrote the manuscript. All authors discussed the findings and approved the final version.

## CONFLICTS OF INTEREST STATEMENT

The authors declare that they have no competing interests.

## FUNDING INFORMATION

This work was supported by a joint research activity by the German Research Foundation (DFG, Deutsche Forschungsgemeinschaft GE 565/9‐1, Project number: 405009566) and the Taiwanese Ministry of Science and Technology (MOST, 107‐2923‐B‐178‐001‐MY3) to GG and YIL, and grants from the Yunnan Academy of Forestry and Grassland, People's Republic of China, to H.J. Funded by the Open Access Publishing Fund of the University of Bayreuth.

## Supporting information


**Fig. S1.** (A) Nitrogen concentration (Total N) for *Yoania amagiensis* var. *squamipes* (n = 3) and *Yoania japonica* (n = 5) and (B) Oxygen stable isotope enrichment factors (ε^18^O) for *Yoania amagiensis* var. *squamipes* (n = 3), and reference plants. The box spans the first and third quartile, while the horizontal line in the box represents the median; whiskers extend to 1.5*interquartile range. Different letters indicate statistically significant differences (Mann–Whitney *U*‐test) between groups.

**Table S1.** Summary of fungal operational taxonomic units (OTUs) and their frequencies detected in mycorrhizal rhizomes using the Illumina Miseq platform.
**Table S2.** Mean and single δ^15^N, δ^13^C, δ^2^H, δ^18^O values, enrichment factors ε^15^N, ε^13^C, ε^2^H, ε^18^O, that is, isotope shifts of individual plants relative to the mean isotopic composition of autotrophic reference plants, and Total N concentration data of autotrophic reference species for the two *Yoania* species collected in Taiwan. Nomenclature follows the WFO (2025): World Flora Online. Published on the Internet.
**Table S3.** Pairwise comparisons ε^13^C, ε^15^N, ε^2^H, ε^18^O and Total N between the two *Yoania* species and autotrophic reference species using the Mann–Whitney *U*‐test with Bonferroni–Holm correction (wilcox_test, R‐package rstatix). Reported are the test statistics W, *P*‐values and the effect size (wilcox_effsize, R‐package rstatix).
**Table S4.** Pairwise comparisons of ε^13^C and ε^15^N of the two *Yoania* species and fully mycoheterotropic orchid species mycorrhizal with wood‐decaying fungi from the literature using Mann–Whitney *U*‐test (wilcox_test, R‐package rstatix). Reported are the test statistics W, *P*‐values and the effect size (wilcox_effsize, R‐package rstatix).

## Data Availability

The data that support the findings of this study are openly available in NCBI BioSample Documentation at https://www.ncbi.nlm.nih.gov/biosample/docs/, reference number SAMN50547615‐50547622.
