# Supplementary figures and images for: Mycorrhizal specificity of fully mycoheterotrophic Yoania in Taiwan and China and novel natural abundance stable isotope patterns
Source: Plant Biol (Stuttg). 2026 Mar 11;28(4):1159–68. doi: 10.1111/plb.70195 (PMC13175952; doi:10.1111/plb.70195)

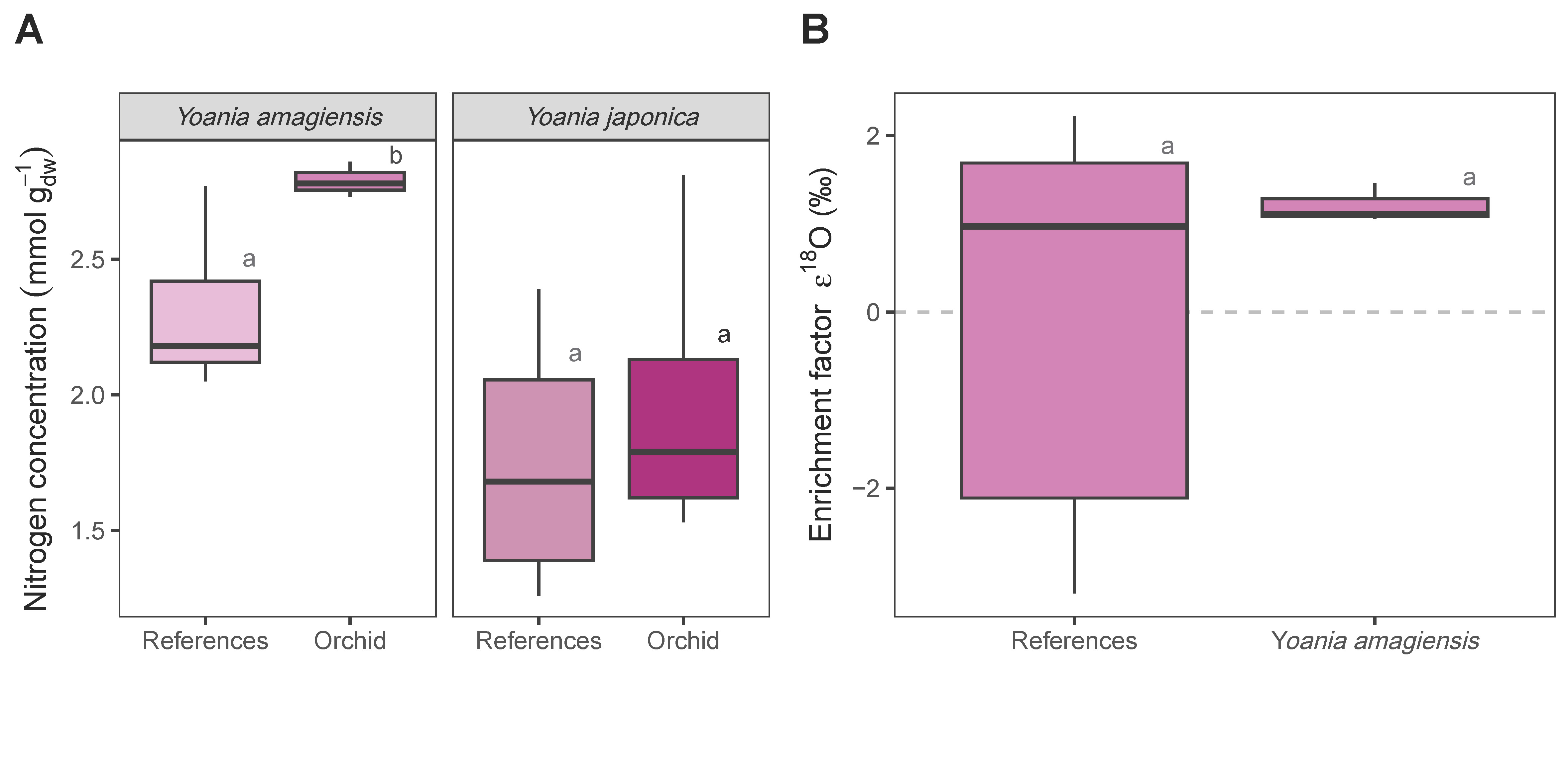

Supplement: Supplementary file 1 — Fig. S1. (A) Nitrogen concentration (Total N) for Yoania amagiensis var. squamipes (n = 3) and Yoania japonica (n = 5) and (B) Oxygen stable isotope enrichment factors (ε18O) for Yoania amagiensis var. squamipes (n = 3), and reference plants. The box spans the first and third quartile, while the horizontal line in the box represents the median; whiskers extend to 1.5*interquartile range. Different letters indicate statistically significant differences (Mann–Whitney U‐test) between groups. [file PLB-28-1159-s001.jpg]
